# Emotion regulation, mindfulness, and self-compassion among patients with borderline personality disorder, compared to healthy control subjects

**DOI:** 10.1371/journal.pone.0248409

**Published:** 2021-03-17

**Authors:** Ella Salgó, Liliána Szeghalmi, Bettina Bajzát, Eszter Berán, Zsolt Unoka

**Affiliations:** 1 Department of Psychiatry and Psychotherapy, Semmelweis University, Budapest, Hungary; 2 Institute of Psychology, Pázmány Péter Catholic University, Budapest, Hungary; Medical University of Vienna, AUSTRIA

## Abstract

**Objectives:**

Emotion regulation difficulties are a major characteristic of personality disorders. Our study investigated emotion regulation difficulties that are characteristic of borderline personality disorder (BPD), compared to a healthy control group.

**Methods:**

Patients with BPD (N = 59) and healthy participants (N = 70) filled out four self-report questionnaires (Cognitive Emotion Regulation Questionnaire, Difficulties in Emotion Regulation Scale, Five Facet Mindfulness Questionnaire, Self-Compassion Scale) that measured the presence or lack of different emotion-regulation strategies. Differences between the BPD and the healthy control group were investigated by Multivariate Analysis of Variance (MANOVA) and univariate post-hoc F-test statistics.

**Results:**

People suffering from BPD had statistically significantly (p<0.05) higher levels of emotional dysregulation and used more maladaptive emotion-regulation strategies, as well as lower levels of mindfulness and self-compassion compared to the HC group.

**Conclusion:**

In comparison to a healthy control group, BPD patients show deficits in the following areas: mindfulness, self-compassion and adaptive emotion-regulation strategies. Based on these results, we suggest that teaching emotion-regulation, mindfulness, and self-compassion skills to patients can be crucial in the treatment of borderline personality disorder.

## 1. Introduction

Emotion regulation consists of the capabilities to process and modulate affective experience. Difficulties with these abilities are often present in people suffering from borderline personality disorder (BPD); moreover, emotion dysregulation is considered a core attribute of this mental disorder [1, 2]. BPD patients are frequently experiencing overwhelming negative emotions such as abandonment, loneliness, jealousy, feeling rejected, hatred, envy, anger, shame and guilt [3–5]. They often report aversive tension, a diffuse, highly aroused state with negative valence [6], and they have difficulties with identifying, naming, or putting into context these emotional states [7–10]. Their reactions to their emotions are often inappropriate: they can be impulsive and have angry outbursts, impulsive behavioral reactions and labile affect. The way they respond to their negative emotions influences the frequency or intensity with which negative affect arises. Their emotion and affect regulation strategies are dysfunctional; for example, they have a tendency towards clinginess [11], dissociation [12], emptiness [13], self-harming behavior [14], alcohol and substance use [15], impulsive sexual behaviors [16], binging, purging [17], and rumination [18]. We hypothesized that they are less able to use functional emotion regulation, such as being mindfully aware of one’s emotions, to label, accept and validate emotions, and to tolerate negative or positive emotion-related distress [2].

In the current study, we aimed to investigate whether a broad range of emotion regulation difficulties are characteristic to BPD patients compared to a healthy control group. We also wanted to examine emotion regulation difficulties, adaptive and maladaptive cognitive emotion regulation strategies, mindfulness, and self-compassion in the two groups. Our study is partly a replication and partly an extension of previous studies.

### 1.1 Difficulties in emotion regulation in BPD

Emotion regulation difficulties are a significant characteristic of BPD [1], such that BPD symptoms and interpersonal problems in BPD are found to be mediated by emotion regulation difficulties [19, 20]. The results of a study suggest that emotion dysregulation, particularly lack of access to emotion regulation strategies and lack of emotional clarity, mediate the relationship between BPD symptoms and poor physical health symptoms (e.g., “headaches,” “dizziness,” “stomach pain”) measured eight months later [21]. A study of 100 adults diagnosed with BPD demonstrated significant reductions in emotion dysregulation (measured by DERS) after a six-month-long dialectical behavior therapy intervention [22]. Emotion dysregulation assessed by DERS explained unique variance in BPD symptoms, showing that impulse control difficulties and limited access to emotion regulation strategies have the strongest relationship to BPD [23, 24]. As a consequence of emotion dysregulation, people suffering from BPD show deficits in action planning and emotion regulation functioning as a mechanism of effective and goal-directed behavior [25]. In our study, we would like to compare emotional dysregulation in the BPD and HC groups in an adult sample by using DERS as a measurement tool for emotion dysregulation. The only previous study [26] that compared BPD and HC groups by using DERS analyzed differences in its "acceptance" subscale only. Our study complements these findings by analyzing all subscales of DERS.

### 1.2 Cognitive emotion regulation in BPD measured by CERQ

Cognitive strategies have a crucial role in emotion regulation. In order to measure adaptive and non-adaptive cognitive emotion regulation strategies, the Cognitive Emotion Regulation Questionnaire (CERQ) [27] has been developed, using the following nine subscales: self-blame, other-blame, rumination or focus on thought, catastrophizing, putting into perspective, positive refocusing, positive reappraisal, acceptance and refocus on planning.

Using CERQ, it has been shown that people with BPD tend to practice maladaptive emotion regulation strategies. A study showed [26] that BPD patients have more frequent use of maladaptive cognitive emotion regulation strategies (suppression, rumination, avoidance) and less frequent use of adaptive strategies (acceptance, cognitive reappraisal, problem-solving) relative to HC. Using CERQ, Wijk-Herbrink, and colleagues [28] identified three higher-order factors; adaptive coping, non-adaptive coping, and external attribution style in people with personality disorders. They found that they used more non-adaptive coping and less adaptive coping strategies as compared to a general population sample. This study suggests that dysfunctional cognitive emotion regulation can be a characteristic of personality disorders in general. Another study, however, shows no significant differences between people of cluster B personality disorders and healthy control in the nine cognitive emotion regulation strategies measured by CERQ [29]. Research found [30] that the use of maladaptive cognitive emotion regulation strategies (self-blame, blaming others, rumination, and catastrophizing) were related to high levels of negative affectivity and increased psychological problems in people with PDs. Another study compared BPD and schizotypal PD, where the participants scored similarly on CERQ, except for the catastrophizing subscale that had higher scores in BPD patients [31]. Our study will have added value to the previous studies comparing BPD and HC groups by using CERQ [26, 29, 32], in as much as our research analyzes all the subscales of CERQ and determines effect sizes as well.

### 1.3 BPD and mindfulness

Mindfulness is a non-judgmental, present-focused state of mind characterized by present-moment awareness, where thoughts, perceptions, and feelings are accepted and purposefully brought into attention [33]. Low levels of mindfulness have been proven to play a significant role in personality psychopathology, and specifically in BPD [34]. Mindfulness is inversely associated with BPD features and core areas of dysfunctionality, such as interpersonal ineffectiveness, impulsive, passive emotion regulation, and neuroticism [35, 36]. In a study exploring differences in the five mindfulness facets (measured by FFMQ) among patients diagnosed with either obsessive-compulsive disorder, major depressive disorder or borderline personality disorder and HC, BPD patients scored lower on all mindfulness facets compared to the HC group [37]. In a study conducted by Nicastro et al. [38] fewer mindfulness skills were found in BPD patients than in control participants. Findings demonstrate that dispositional mindfulness is negatively associated with BPD features and suicidal thinking among patients in substance use treatment [39]. The inverse relation between BPD and mindfulness can be explained by the difficulties of BPD patients to be consciously aware of their experiences in the present moment instead of focusing on general concepts. The latter may impair their ability to effectively regulate their emotions [40].

Mindfulness is a multidimensional construct. Yu and Clark [36] investigated the relationship between mindfulness (assessed by FFMQ) and borderline personality traits in a non-clinical sample and found that mindfulness facets relate differentially to BPD symptoms, among them "non-judging" is the facet most strongly related to BPD traits. Research suggests that for BPD patients, mindful self-observation can be an adaptive alternative to rumination when feeling angry [32].

Conceptual integration of mindfulness and emotion regulation was proposed by Chambers, Gullone, and Allen [41]. According to their review, cognitive emotion regulation strategies and mindfulness fundamentally differ in that according to the concept of emotion regulation, unpleasant thoughts/appraisals need to be acted upon or manipulated in some way to make them less distressing. In contrast, mindfulness considers all mental phenomena as mere mental events that do not need to be transformed. Their proposed "mindful emotion regulation" is the capacity to remain mindfully aware of the experienced emotions, irrespective of their valence, intensity, and without attempting to reappraise or modify them. Based on this proposition, in our study, we consider mindfulness a potential form of emotion regulation. Our study’s additional value to the previous research comparing BPD and HC groups by exploring the five mindfulness facets [37] is that it evaluates the effect sizes in terms of the magnitude of the difference between the two groups.

### 1.4 BPD and self-compassion

Self-compassion is a self-regulation strategy that counters self-criticism and related negative self-directed emotions, such as shame [42]. Neff [43] conceptualized self-compassion with the following three dimensions: a) self-kindness vs. self-judgment, b) common humanity vs. isolation, and c) mindfulness vs. over-identification. Based on a quantitative meta-analytic study, each of these factors are suggested to assist adaptive self-regulatory processes [44]. One may reason that such self-regulatory processes in general—including emotion-regulation—are impaired in BPD since BPD is frequently associated with childhood trauma and abuse [45–47], and childhood trauma exposure and emotional dysregulation are suggested to have a complex and bidirectional relationship [48]. Linehan’s biosocial theory [49] suggests that what she calls "invalidating environments" during childhood may play an important role in the subsequent development of BPD in adolescence, by hindering the development of self-compassion and emotion-regulation. However, a study [50] found that even though childhood parental invalidation and lack of self-compassion are both strongly associated with BPD symptoms, their associations with BPD symptoms are independent of each other. In contrast, traumatic experiences may contribute to a self-invalidating and self-critical cognitive style [49]. Other studies suggest that self-criticism is a diagnostic element [51] and a frequent characteristic of BPD [52–54].

Research shows that loving-kindness and compassion meditation based on self-compassion lowers self-criticism and improves self-kindness and acceptance in BPD patients [53]. Moreover, self-compassion seems to mediate between mindfulness and BPD symptoms, and between mindfulness and emotion dysregulation as well [55]. Self-compassion is also considered the outcome of mindfulness practice [56].

The above studies suggest that the lack of self-compassion is associated with BPD symptoms and that improved self-compassion can ease the emotional pain experienced in BPD. Some research has already been conducted on comparing BPD population to HC in the context of self-compassion, although with a different aim. Scheibner and colleagues [55] used the Self Compassion Scale (SCS) to compare BPD patients with HC, and found significant differences between these two groups in terms of self-compassion. A study found that BPD patients had significantly higher fears and resistances to all forms of compassion (fears of self-compassion, fears of being open to compassion of others, fears of being compassionate to others) compared to the control group [57]. The current study is an extension of the previous one that compared BPD and HC groups by using SCS [55] since it investigates group differences in the SCS subscales as well.

### 1.5 Mini review of the literature of the studies that compared BPD and HC on one of the following scales: CERQ, DERS, FFMQ, and SCS

Why do we need one further study? As outlined in the Introduction, there are several studies examining emotion regulation difficulties in BPD. However, there are only a few studies comparing adult BPD groups to healthy control participants, and those that exist do not examine CERQ, DERS, FFMQ and SCS simultaneously by analyzing all of their subscales. We prepared a summary of the literature that compares adult BPD and HC groups by using CERQ, DERS, FFMQ and/or SCS (see Table 1). By administering these four questionnaires in the two groups in the current study, we cover a more comprehensive array of emotion regulation strategies than previous studies.

**Table 1:** Mini review of the literature comparing BPD and HC groups based on emotion regulation strategies/dysfunctionalities.

Authors, date	Title	Source	Sample	Used scales	Findings	Effect sizes—BPD vs. HC	Subscale analysis
Daros et al., 2018	Cognitive Emotion Regulation Strategies in Borderline Personality Disorder: Diagnostic Comparisons and Associations with Potentially Harmful Behaviors	Psychopathology	BPD (n = 30) MAD (n = 30) HC (n = 32)	**CERQ**, CSI, DASS, **DERS**, MEAQ, RRS, WBSI	BPD subjects endorsed more maladaptive cognitive ER strategies and fewer adaptive strategies compared to HC. Compared to MAD subjects, BPD individuals endorsed more maladaptive cognitive ER strategies, but only when those with subthreshold BPD symptoms in the MAD group were excluded.	**Cohen’s d**: CERQ pos refocus: -0.52 CERQ pos reappraisal: -1.52 CERQ putting into persp: -0.56 CERQ refocus on planning: -1.30 CERQ acceptance: -0.05 DERS acceptance (reversed): -2.20	5 subscales of CERQ and 1 of DERS were utilized.
Didonna et al. 2019	Relations of mindfulness facets and psychological symptoms among individuals with a diagnosis of Obsessive-Compulsive Disorder, Major Depressive Disorder and Borderline Personality Disorder	Psychology and Psychotherapy Theory Research and Practice	OCD (n = 55), MDD (n = 50), BPD (n = 48), HC (n = 50)	BDI-II, DES, **FFMQ**, SCL-90, TAS-20	Mindfulness abilities seem to be impaired in psychiatric patients compared with HC. There are disease-specific relationships between some mindfulness facets and specific psychological variables	**-**	yes
Heidari et al., 2015	Comparative Evaluation of Cognitive Emotion Regulation between "B" Personality Disorders and Normal Persons	Procedia—Social and Behavioral Sciences	AsPD (n = 46) BPD (n = 46) HPD (n = 46) NPD (n = 46) HC (n = 46)	**CERQ**, MCMI III,	There were no significant differences between people of cluster B personality disorders and people of normal personality in nine cognitive coping strategies of CERQ.	**Partial Eta Squared**: CERQ Self-blame: .000 Acceptance: .005 Rumination: .000 Pos refocus: .008 Refocus on planning: .032 Pos reappraisal: .000 Putting into persp: .006 Catastrophizing: .022 Blaming others: .063	yes
Sauer et al., 2016	Emotion regulation choice in female patients with borderline personality disorder: Findings from self-reports and experimental measures	Psychiatry Research	BPD (n = 24) MD (n = 19) HC (n = 32)	BSL-23, BDI- II, **CERQ**, RSQ-D, SCL-9,	Both patient groups showed maladaptive self-reported emotion regulation choice profiles compared with HC. No differences between the groups in the choice of distraction and reappraisal on the behavioral level and in heart rate responses. In BPD, within-group analyses revealed a positive correlation between symptom severity and the preference for distraction under high-intensity borderline-specific stimuli.	**-**	yes
Scheibner et al., 2017	Self-Compassion Mediates the Relationship Between Mindfulness and Borderline Personality Disorder Symptoms	Journal of Personality Disorders	BPD (n = 29) HC (n = 30)	FFA, **SCS**	Self-compassion mediates the relationship between mindfulness and BPD symptom severity as well as between mindfulness and emotion dysregulation	**Kappa-squared**: k2 = .20 (Effect size of the indirect effect of mindfulness on BPD symptom severity via self-compassion.)	no

**Notes**: AsPD = Antisocial Personality Disorder, BPD = Borderline Personality Disorder, BSL-23 = Borderline Symptom List, BDI-II = Beck Depression Inventory, CERQ = Cognitive Emotion Regulation Questionnaire, CSI = Coping Strategies Inventory, DASS = Depression, Anxiety and Stress Scale, DERS = Difficulties in Emotion Regulation Questionnaire, DES = Dissociative Experience Scale, FFA = Freibruger Fragebogen zur Achtsamkeit (Freiburg Mindfulness Inventory), HC = healthy control, HPD = Histrionic Personality Disorder, MAD = mixed anxiety and/or depressive disorder, MCMI III = Millon Clinical Multiaxial Inventory, MD = Major Depression, MEAQ = Multidimensional Experiential Avoidance Questionnaire, NPD = Narcissistic Personality Disorder, RRS = Ruminative Response Style Questionnaire, RSQ-D = Response Style Questionnaire (German version), SCL-9 = Symptoms Checklist 9, SCS = Self-Compassion Scale, TAS-20 = Toronto Alexithymia Scale, WBSI = White Bear Suppression Inventory.

### 1.6 Hypothesis

We hypothesized that the BPD and HC groups would show significant differences in terms of emotion regulation, mindfulness, and self-compassion. Furthermore, dysfunctional emotion regulation strategies and lack of self-compassion would be predominant among BPD patients. We also hypothesized that adaptive emotion regulation strategies, mindfulness skills, and self-compassion techniques would score higher in the HC group.

## 2. Method

### 2.1 Subjects and procedure

Subjects participated in a four-week-long inpatient psychotherapy program at Semmelweis University’s Department of Psychiatry and Psychotherapy between 2017 and 2019. Psychiatrists and clinical psychologists made the diagnoses during intake interviews. Data has been gathered from 59 subjects diagnosed with borderline personality disorder and from 70 healthy control subjects. Medical students recruited age, gender, and education matched healthy control volunteers who were acquaintances and relatives of university students with no known psychiatric disorders. There were 104 female (80.6%) and 25 male (19.4%) participants, with a mean age of 30.7 years (SD = 11.1, range = 18–57). Regarding educational level, 0% completed just the first six years of primary school, 28.7% passed A-level exams, 24.8% did not obtain A-level exams, 3.8% dropped out of college, 9.3% completed vocational studies, 11.6% obtained a college degree, 8.5% dropped out of the university while 13.1% obtained university degree. (To see the distribution of clinical diagnosis, see Table 2).

**Table 2:** Sociodemographic and clinical variables of patients with borderline personality disorder and healthy comparison subjects.

	Groups				
Characteristics	Borderline Personality disorders (N = 59)		Healthy Control (N = 70)		Test-statistic
	*Mean*	*SD*	*Mean*	*SD*	*F (x*,*y)*
**Age**	30.2	10	31.2	12	0.2 (1,127)
	**N**	**%**	**N**	**%**	**χ2**
**Gender**					1.2
Male	9	15.3	16	22.9	
Female	50	84.7	54	77.1	
**Education**					9.9
1. first 6 years of primary school	0	0	0	0	
2. A-levels	18	30.5	19	27.1	
3. without A-levels	11	18.6	21	30	
4. dropped out of college	3	5.1	2	2.9	
5. completed vocational studies	9	15.3	3	4.3	
6. obtained college degree	5	8.5	10	14.3	
7. dropped out of university	3	5.1	8	11.4	
8. obtained university degree	10	16.9	7	10	
**Types of personality disorders**					
Paranoid	8	13.6	0	0	
Borderline	59	100	0	0	
Histrionic	6	10.2	0	0	
Narcissistic	2	3.4	0	0	
Avoidant	25	42.4	0	0	
Dependent	15	25.4	0	0	
Obsessive-compulsive	14	23.7	0	0	
Passive-Aggressive	8	13.6	0	0	
Depressive	26	44.1	0	0	
Schizoid	1	1.7	0	0	
Schizotypal	3	5.1	0	0	
**Comorbid disorders**					
Depressive episode	18	40	0	0	
Generalized Anxiety disorders	16	35.6	0	0	
Bipolar disorder	21	46.7	0	0	
Panic disorder	3	6.7	0	0	
PTSD	0	0	0	0	
OCD	1	2.2	0	0	
Psychotic disorder	4	8.9	0	0	
Substance use disorder	5	11.1	0	0	
Eating disorder	7	15.6	0	0	
Somatoform disorder	1	2.2	0	0	

Notes: * p < 0.05. ** p < 0.01.

Subjects had been provided with sufficient information about the research and signed an informed consent sheet. Their anonymity was guaranteed. Participants were diagnosed with SCID II interviews and filled out questionnaires online. The Regional and Institutional Committee of Science and Research Ethics of Semmelweis University approved the research procedure.

### 2.2 Self-reported questionnaires measuring emotion regulation strategies

**The Cognitive Emotion Regulation Questionnaire (CERQ)** is a 36-item questionnaire measuring cognitive emotion regulation strategies applied after having experienced negative life events or situations [27]. It assesses nine cognitive emotion regulation strategies: self-blame, other-blame, rumination, or focus on thought, catastrophizing, putting into perspective, positive refocusing, positive reappraisal, acceptance, and refocus on planning. Cronbach’s α coefficients of the subscales in this study ranged between.60 (acceptance) and.89 (positive refocusing). Cognitive emotion regulation strategies were measured on a 5-point Likert scale ranging from 1 (almost never) to 5 (almost always). The Hungarian version of the questionnaire had been validated by Miklósi and colleagues [58].

**The Difficulties in Emotion Regulation Scale (DERS)** [59], was created based on four main aspects of emotion regulation, as defined by the authors:

“(a) awareness and understanding of emotions,
(b) acceptance of emotions,
(c) ability to control impulsive behaviors and behave in accordance with desired goals when experiencing negative emotions,
(d) ability to use situationally appropriate emotion regulation strategies flexibly to modulate emotional responses as desired, in order to meet individual goals and situational demands.” (pp42).

Higher scores on the measure indicate greater dysfunctionality or dysregulation. DERS was implemented [59] in its Hungarian version [60] in order to determine the degree of difficulty in emotion regulation. The 36 items of DERS are organized into a 6-factor structure: non-acceptance of emotional responses, difficulty engaging in goal-directed behavior, impulse control difficulties, lack of emotional awareness, limited access to emotion regulation strategies and lack of emotional clarity. Cronbach’s α coefficients of the DERS subscales in this research ranged between.67 (impulse control difficulties) and.91 (limited access to emotion regulation strategies). DERS’s scales are rated on a 5-point Likert scale.

**The Self-Compassion Scale (SCS)**, developed by Dr. Kristine Neff [43], is applied to measure self-compassion, which is defined as compassion turned inward and refers to how we relate to ourselves in instances of perceived failure, inadequacy or personal suffering [61]. The scale consists of 26 items rated on a 5-point Likert scale. Its three subscales are self-kindness versus self-judgment, a sense of common humanity versus isolation, and mindfulness versus over-identification. Cronbach’s α coefficients of the subscales in this study ranged between.56 (self-judgment) and.84 (self-kindness). The Hungarian version of SCS was implemented by Sági and co-workers [62]. In our study, we interpret our findings according to the two-factor model of SCS, which collapses self-kindness, common humanity, and mindfulness items into a positive, "self-compassion" factor and self-judgment, isolation, and over-identification items into a negative, "self-criticism" factor [61].

**The Five Facet Mindfulness Questionnaire** includes 39 items that examine the five major aspects of mindfulness on a 5-point Likert scale: observation, description, mindful actions, non-judgmental inner experience and non-reactivity [63]. Cronbach’s α coefficients of the subscales in this study ranged between.70 (non-reactivity) and.88 (description). The Hungarian adaptation of the scale was carried out by Józsa (unpublished work).

### 2.3 Statistical analysis

Our statistical analyses tested the hypothesis that difficulty of emotion regulation scores are higher in patients with borderline personality disorder than in healthy participants against the null-hypothesis of no difference. The differences between the BPD and HC groups in terms of their DERS, CERQ, FFMQ and SCS sub-scales were investigated by Multivariate Analysis of Variance (MANOVA), and subsequently by post-hoc univariate F-test statistics determined from the MANOVA analysis.

The analyses were conducted based on a hierarchical approach. Specifically, first, in our primary analysis, the total score on each of the four scales of interest was tested. Study group (BPD or HC) was used as the independent variable in the MANOVA, whereas DERS-total, CERQ adaptive emotion regulation total, CERQ maladaptive emotion regulation total, FFMS-total, and SCS-total scales served as dependent variables. Second, in case the primary analyses yielded a significant difference, we conducted post-hoc analyses by determining the univariate F-statistics to examine the differences between the two groups in the subscales of the four scales mentioned above. In the post-univariate analyses, we used the Hochberg correction to adjust for the inflation of alpha error as a result of multiple testing. We added an asterisk to those results that remained statistically significant after correction for multiple testing in the tables.

Because of different sample sizes, effect sizes were measured by Hedges’ g [64], which provides a measure of effect size weighted according to the relative size of each sample (small effect = 0.2, medium effect = 0.5, large effect = 0.8, [65]). In order to assess the homogeneity of variances, Levene’s test was performed. Where Levene’s test indicated unequal variances, a Welch test was performed.

Based on the adopted statistical approach (MANOVA), we conducted a statistical power analysis for our primary comparisons to determine the assay sensitivity (i.e., the statistical effect size for a detectable group difference) in the study The power analysis followed the procedure described in the literature [66, 67]. The input parameters for the computation were the available sample size (n = 59 and 70 in the two groups, respectively), and the required alpha threshold level (= 0.05) and level of correlation in terms of Pearson’r among the individual variables used in the MANOVA analysis. Since the individual measures used in the MANOVA are expected to be correlated for Pearson’s we conservatively we adopted a value of 0.5 (i.e., 25% in terms of overlapping variance). Our results indicated that the available sample size provides >80% power to detect a standardized group difference of 0.3 on the variables entered in the MANOVA analysis; this value is considered a small effect size, and was deemed to provide sufficient assay sensitivity for the study.

## 3. Results

### 3.1. Demographic, descriptive and clinical characteristics

The current study included a sample of 129 participants (BPD = 59 (9 males), HC = 70 (16 males)). The two groups did not differ significantly on gender (chi-square test: χ2 = 1.2, p = 0.27) in levels of education (chi-square test: χ2 = 9.9, p = 0.12) or in age (ANOVA: (F (1,127) = 0.2; p = 0.62). See Table 2.

### 3.2 MANOVA for the total scores

We conducted MANOVA multivariate statistics to determine whether differences between the means of the BPD and HC groups are statistically significant based on the scales’ total scores. The primary MANOVA of the total scores of DERS, CERQ Adaptive, CERQ Non-Adaptive, FFMQ, and SCS found statistically significant differences between the BPD and the HC groups: Multivariate F (5,123) = 61.24, p < .0001; Wilk’s Λ = 0.29. Results of the post-hoc univariate comparisons are presented in Table 3.

**Table 3:** Group comparisons for the total scores of the four scales.

Variable	Total Standard Deviation	Pooled Standard Deviation	Between Standard Deviation	R-Square	R-Square / (1-RSq)	F value^a^	Pr > F	g
DERS Total	0.7986	0.5092	0.8691	0.5967	1.4795	187.90	< .0001	2.428
CERQ Adaptive	0.7179	0.5488	0.6555	0.4201	0.7245	92.02	< .0001	-1.683
CERQ Non-Adaptive	0.6947	0.5469	0.6073	0.3851	0.6263	79.54	< .0001	1.589
FFMQ Total	0.5082	0.3619	0.5046	0.4968	0.9874	125.40	< .0001	-2.002
SCS Total	0.5040	0.2922	0.5796	0.6665	1.9983	253.78	< .0001	-2.870

Notes:

^a^: Univariate post-hoc F-test statistics determined from the MANOVA analysis.

g: Effect size measured by Hedge’s g formula.

### 3.3 MANOVA of the two groups based on the difficulty of emotion regulation

Since the primary analyses of DERS total score yielded a significant difference, we conducted post-hoc analyses to examine the differences between the two groups in the subscales of the DERS. In every subscale of DERS, patients with BPD had higher scores than healthy participants (DERS total F(1,127) = 187.90, *p* < 0.001). Effect sizes between the BPD and the HC groups are large, except for one medium effect size in the lack of emotional awareness subscale. Results are presented in Table 4.

**Table 4:** Group comparisons for the BPD and HC groups on the subscale scores of the difficulties of emotion regulation scale, and effect sizes measured by Hedge’s g formula.

Measure	Diagnostic groups				Difference among diagnostic groups			
DERS	BPD (N = 59)		HC (N = 70)		F^a^	df	*p*	*g*
	*Mean*	*SD*	*Mean*	*SD*				
**non-acceptance**	3.04	1.02	1.91	0.81	48.69	1,127	< 0.001*	1.250
**difficulty engaging in goal-directed behavior**	3.78	0.83	2.44	0.77	90.12	1,127	< 0.001*	1.679
**impulse control difficulties**	3.37	0.95	1.86	0.66	111.03^W^	1,127	< 0.001*	1.874
**lack of emotional awareness**	3.01	0.83	2.44	0.77	16.08	1,127	< 0.001*	0.714
**lack of emotional clarity**	2.82	0.95	1.79	0.70	49.32^W^	1,127	< 0,001*	1.250
**limited access to emotion regulation strategies**	3.75	0.81	1.93	0.65	197.16	1,127	< 0.001*	2.501

Notes:

^a^: Univariate post-hoc F-test statistics determined from the MANOVA analysis.

g: Effect size measured by Hedge’s g formula; BPD = patients with borderline personality disorder (1); HC = healthy control (2);

^W^: where Levene’s test indicated unequal variances a Welch test was performed. Results that remained statistically significant after correction for multiple testing were marked with an asterisk.

Both the primary analyses of “adaptive emotion regulation total” and “maladaptive emotion regulation total” scores yielded a significant difference; we conducted post-hoc analyses to examine the differences between the two groups in the subscales of the CERQ. Only its two subscales, “other-blame” and “acceptance,” did not show significant differences between the two groups. Maladaptive emotion regulation strategies scored higher in the BPD group, while adaptive strategies scored higher in the HC group. (CERQ adaptive total F(1,127) = 92.02, p< 0.001, CERQ maladaptive total F(1,127) = 79.54, p< 0.001). Large effect sizes were found between the BPD and HC groups, with the exception of the other-blame and acceptance scales. Negative effect sizes indicate poorer results on the given subscale in the BPD group, e.g., putting into perspective. Results are presented in Table 5.

**Table 5:** Group comparisons of the BPD and HC groups on the subscale scores of the cognitive emotion regulation questionnaire, and effect sizes measured by Hedge’s g formula.

Measure	Diagnostic groups				Difference among diagnostic groups			
CERQ	BPD (N = 59)		HC (N = 70)		F^a^	df	*p*	*g*
	*Mean*	*SD*	*Mean*	*SD*				
**self-blame**	3.75	0.82	2.46	0.79	81.07	1,127	< 0.001*	1.594
**other-blame**	1.94	0.80	1.79	0.57	1.58^W^	1,127	0.22	0.204
**rumination**	3.28	0.90	2.48	0.88	25.75	1,127	< 0.001*	0.899
**catastrophizing**	2.95	1.05	1.74	0.73	58.38^W^	1,127	< 0.001*	1.358
**putting into perspective**	2.30	0.76	2.98	0.80	24.58	1,127	< 0.001*	-0.869
**positive refocusing**	1.64	0.58	3.08	0.92	105.62^W^	1,127	< 0.001*	-1.838
**positive reappraisal**	2.20	0.81	3.77	0.85	111.82	1,127	< 0.001*	-1.874
**acceptance**	2.55	0.64	2.56	0.65	0.01	1,127	0.911	-0.015
**refocus on planning**	2.89	1.03	3.84	0.91	30.25	1,127	< 0.001*	-0.972

Notes:

^a^: Univariate post-hoc F-test statistics determined from the MANOVA analysis.

g: Effect size measured by Hedge’s g formula; BPD = patients with borderline personality disorder (1); HC = healthy control (2);

^W^: where Levene’s test indicated unequal variances a Welch test was performed. Results that remained statistically significant after correction for multiple testing were marked with an asterisk.

The FFMQ total score’s primary analyses yielded a significant difference (FFMQ total F(1,127) = 125.40, p < 0.001), so we conducted post-hoc analyses to examine the differences between the two groups in its subscales. Four subscales; "mindful actions", "non-judgmental inner experience", "non-reactivity" and "description" had higher scores in the HC group than in the BPD group. Only the "observation" subscale did not present significant differences between the two groups. Effect sizes are medium to large between the two groups, with the exception of the observation subscale that yielded very small effect sizes among the groups. Results are presented in Table 6.

**Table 6:** Group comparisons of the BPD and HC groups on the subscale scores of the five factor mindfulness questionnaire, and effect sizes measured by Hedge’s g formula.

Measure	Diagnostic groups				Difference among diagnostic groups			
FFMQ	BPD (N = 59)		HC (N = 70)		F	df	*p*	g
	Mean	SD	Mean	SD				
**observation**	2.93	0.79	2.94	0.71	0.00	1,127	0.962	-0.013
**mindful actions**	2.95	0.62	3.98	0.64	82.76	1,127	< 0.001*	-1.616
**non-judgmental inner experience**	2.63	0.65	3.86	0.70	102.95	1,127	< 0.001*	-1.800
**non-reactivity**	2.31	0.57	2.79	0.66	19.24	1,127	< 0.001*	-0.789
**description**	3.01	1.01	3.86	0.68	31.28^W^	1,127	< 0.001*	-1.003

Notes:

a: Univariate post-hoc F-test statistics determined from the MANOVA analysis.

g: Effect size measured by Hedge’s g formula; BPD = patients with borderline personality disorder (1); HC = healthy control (2);

^W^: where Levene’s test indicated unequal variances a Welch test was performed. Results that remained statistically significant after correction for multiple testing were marked with an asterisk.

The primary analyses of SCS total score yielded a significant difference, so we conducted post-hoc analyses to examine the differences between the two groups in its subscales. The relevant subscale-pairs in SCS present opposing trends in their mean scores; “self-kindness,” “common humanity,” and “mindfulness” scored higher in the HC group, while “self-judgment,” “isolation,” and “over-identification” have higher scores in the BPD group. (SCS positive subscales total F(1,127) = 82.55, p< 0.001, SCS negative subscales total F(1,127) = 234.00, p< 0.001). Effect sizes are large between the BPD and HC groups. Results are presented in Table 7.

**Table 7:** Group comparisons of the BPD and HC groups on the subscale scores of the self-compassion scale, and effect sizes measured by Hedge’s g formula.

Measure	Diagnostic groups				Difference among diagnostic groups			
SCS	BPD (N = 59)		HC (N = 70)		F^a^	df	*p*	g
	Mean	SD	Mean	SD				
**SCS positive subscales total**	2.21	0.64	3.27	0.66	82.55	1,127	< 0.001*	-1.58
**SCS negative subscales total**	3.95	0.53	2.25	0.69	234.00	1,127	< 0.001*	2.691
**self-kindness**	1.89	0.78	3.24	0.88	82.32	1,127	< 0.001*	-1.606
**self-judgment**	3.89	0.69	2.34	0.86	121.12	1,127	< 0.001*	1.959
**common-humanity**	2.12	0.73	3.19	0.93	51.00	1,127	< 0.001*	-1.266
**isolation**	3.97	0.72	2.18	0.71	197.02	1,127	< 0.001*	2.504
**mindfulness**	2.63	0.73	3.37	0.70	33.74	1,127	< 0.001*	-1.036
**over-identification**	3.98	0.60	2.23	0.74	207.22^W^	1,127	< 0.001*	2.574

Notes:

^a^: Univariate post-hoc F-test statistics determined from the MANOVA analysis.

g: Effect size measured by Hedge’s g formula; BPD = patients with borderline personality disorder (1); HC = healthy control (2);

^W^: where Levene’s test indicated unequal variances a Welch test was performed. Results that remained statistically significant after correction for multiple testing were marked with an asterisk.

## 4. Discussion

Our study has investigated emotion-regulation, mindfulness, and self-compassion abilities in BPD, compared to HC. Results confirmed our hypothesis that people suffering from BPD had a higher level of emotional dysregulation and used more maladaptive emotion-regulation strategies and less adaptive emotion regulation strategies, lower mindfulness and self-compassion levels than HC participants. We are going to discuss each result in detail below.

### 4.1 DERS

In agreement with our hypothesis, results revealed that BPD patients had higher overall emotion dysregulation compared to the HC group. All the six subscales of DERS presented significant differences between the two groups. This result is different from Ibraheim and co-worker’s findings in an adolescent sample, where only two subscales ("limited access to strategies" and "impulse control difficulties") differed significantly [24]. The finding is also in agreement with the results of a meta-analysis by Daros and Williams [2]. In this study, results are based on 93 unique studies indicating that symptoms of BPD were associated with less frequent use of adaptive emotion regulation strategies (i.e., problem solving and cognitive reappraisal) and more frequent use of strategies that are less effective in reducing negative affect (i.e. suppression, rumination, and avoidance).

### 4.2 CERQ

Our results show that the BPD and HC populations have significant differences in almost all CERQ subscales-except for "other-blame" and "acceptance". These results are in harmony with a study [68] examining people with BP features after negative mood and rumination induction. Those participants who scored higher on BP features (measured by Morey’s Personality Assessment Inventory-Borderline Features Scale [69]) reported higher levels of self-blame. Moreover, self-blame, as well as other-blame seemed to be an indicator of impulsive behavior as well [70]. Social exclusion was also associated with self-blame in BPD patients [71]. Another study shows that self-blame partially mediates the relationship between child maltreatment and later non-suicidal self-injury [72].

Our results demonstrate that the inability to put an unpleasant event into perspective is characteristic of the BPD group. This finding is affirmed by the alternative DSM-5 Model of personality disorders [73] which characterized PDs by impairments in personality functioning and pathological personality traits. The incapability of considering and understanding different perspectives is a defining component of the "empathy" factor of the Levels of Personality Functioning Scale, and a proposed diagnostic criteria for BPD.

### 4.3 Mindfulness

Our findings show impaired mindfulness abilities on four mindfulness facets among BPD patients compared to HC; mindful actions, description, non-reactivity and non-judgmental inner experience. The latter subscale presented the largest difference between the BPD and the HC groups. These results are in agreement with previous studies [42, 74, 75]. The result that the "observing" subscale was not significantly different among the three groups is similar to the finding of Didonna and co-worker’s study [37]. Results are in line with the theoretical assumptions that mindfulness practice promotes adaptive emotion regulation strategies [76, 77].

### 4.4 Self-compassion

According to our study, BPD patients scored lower on the adaptive, and higher on the maladaptive dimensions of the self-compassion scale than the healthy control group. Self-compassion has already been examined in BPD in contrast to a healthy population [55, 57]; their findings were similar to our results. A study, where self-compassion was examined in cluster C personality disorders before and after a short-term dynamic psychotherapy, showed that levels of self-compassion increased due to therapy, and this in turn predicted decrease in psychiatric symptoms, and personality pathology [78]. The study of Castilho and co-workers [79] found similar results about self-compassion when examining different clinical samples with diagnoses associated with difficulties in emotion- regulation (e.g. personality disorders).

### 4.5 Limitations

One of the limitations of our study is that self-administered questionnaires might have distorted the data, because self-awareness and self-reflection are impaired functions in BPD [80]. Furthermore, our BPD sample consists of patients participating in a 4 week-long psychotherapy program, suffering from severe symptoms and dysfunctionality; this limits our findings’ generalizability to BPD patients who are functioning better or less motivated to seek help. In both of our samples, the number of female participants is much higher than the number of men. This difference reflects a general observation that BPD is diagnosed predominantly (75%) in females in the clinical sample [81], although Grant et al. did not find gender differences in their epidemiologic survey [82]. The differential gender prevalence of BPD in our clinical setting may be the result of clinical sampling bias. In addition, our sample represents BPD patients who seek pharmaco- and psychotherapeutic help, and this is more characteristic to female BPD patients [83].

## 5. Conclusion

In summary, we can conclude that BPD features have a strong association with emotion dysregulation, and that this manifests in emotion regulation strategies—an increased number of maladaptive ones and a decreased number of adaptive ones—as well as in low levels of mindfulness and self-compassion as compared to an HC group. Based on these results, we suggest that teaching emotion-regulation, mindfulness, and self-compassion skills to patients can be crucial in the treatment of borderline personality disorder.

## Supporting information

S1 File Dataset to analyze BPD and HC groups based on CERQ, DERS, FFMQ and SCS. (XLSX)

## References

[1] Linehan MM, Davison GC, Lynch TR, Sanderson C. Technique factors in treating personality disorders. In: Castonguay LG, Beutler LE, editors. Principles of therapeutic change that work. Oxford, UK: Oxford University Press; 2006. pp. 239–252.

[2] Daros AR, Williams GE. A Meta-analysis and Systematic Review of Emotion Regulation Strategies in Borderline Personality Disorder. Harv Rev Psychiatry. 2019; 27(4):217–232. 10.1097/HRP.0000000000000212 31219881

[3] Zanarini MC, Frankenburg F, DeLuca CJ, Hennen J, Khera GS, Gunderson JG. The pain of being borderline: Dysphoric states specific to borderline personality disorder. Harv Rev Psychiatry. 1998; 6:201–207. 10.3109/10673229809000330 10370445

[4] Reisch T, Ebner-Priemer UW, Tschacher W, Bohus M, Linehan MM. Sequences of emotions in patients with borderline personality disorder. Acta Psychiatr Scand. 2008; 118(1):42–48. 10.1111/j.1600-0447.2008.01222.x 18582346

[5] Unoka Z, Vizin G. To see in a mirror dimly. The looking glass self is self-shaming in borderline personality disorder. Psychiatry Res. 2017; 258:322–329. 10.1016/j.psychres.2017.08.055 28865721

[6] Stiglmayr CE, Grathwol T, Linehan MM, Ihorst G, Fahrenberg J, Bohus M. Aversive tension in patients with borderline personality disorder: A computer-based controlled field study. Acta Psychiatr Scand. 2005; 111(5):372–379. 10.1111/j.1600-0447.2004.00466.x 15819731

[7] Levine D, Marziali E, Hood J. Emotion processing in borderline personality disorders. J Nerv Ment Dis. 1997; 185:240–246. 10.1097/00005053-199704000-00004 9114809

[8] Leible T, Snell W. Borderline personality disorder and multiple aspects of emotional intelligence. Personal Individ Differ. 2004; 37:393–404.

[9] Suvak MK, Litz BT, Sloan DM, Zanarini MC, Barrett LF, Hofmann SG. Emotional granularity and borderline personality disorder. J Abnorm Psychol. 2011; 120(2):414–426. 10.1037/a0021808 21171723 PMC3104325

[10] Conklin C. Z., Bradley B., Westen D. Affect Regulation in Borderline Personality Disorder. J Nerv Ment Dis. 2006; 194(2):69–77. 10.1097/01.nmd.0000198138.41709.4f 16477183

[11] Bornstein RF, Becker-Matero N, Winarick DJ, Reichman AL. Interpersonal dependency in borderline personality disorder: Clinical context and empirical evidence. J Personal Disord. 2010; 24(1):109–127. 10.1521/pedi.2010.24.1.109 20205501

[12] Scalabrini A, Cavicchioli M, Fossati A, Maffei C. The extent of dissociation in borderline personality disorder: a meta-analytic review. J Trauma Dissociation. 2017; 18(4):522–543. 10.1080/15299732.2016.1240738 27681284

[13] Klonsky ED. What is emptiness? Clarifying the 7th criterion for borderline personality disorder. J Personal Disord. 2008; 22(4):418–426. 10.1521/pedi.2008.22.4.418 18684053

[14] Kleindienst N, Bohus M, Ludäscher P, Limberger MF, Kuenkele K, Ebner-Priemer UW, et al. Motives for nonsuicidal self-injury among women with borderline personality disorder. J Nerv Ment Dis. 2008; 196(3):230–236. 10.1097/NMD.0b013e3181663026 18340259

[15] Axelrod SR, Perepletchikova F, Holtzman K, Sinha R. Emotion regulation and substance use frequency in women with substance dependence and borderline personality disorder receiving dialectical behavior therapy. Am J Drug Alcohol Abuse. 2011; 37(1):37–42. 10.3109/00952990.2010.535582 21091162 PMC3052760

[16] Mangassarian S, Sumner L, O’Callaghan E. Sexual impulsivity in women diagnosed with borderline personality disorder: A review of the literature. Sex Addict Compulsivity. 2015; 22(3):195–206.

[17] Selby EA, Doyle P, Crosby RD, Wonderlich SA, Engel SG, Mitchell JD, et al. Momentary emotion surrounding bulimic behaviors in women with bulimia nervosa and borderline personality disorder. J Psychiatr Res. 2012;46(11):1492–1500. 10.1016/j.jpsychires.2012.08.014 22959165 PMC3635132

[18] Martino F, Caselli G, Berardi D, Fiore F, Marino E, Menchetti M, et al. Anger rumination and aggressive behaviour in borderline personality disorder. Personal Ment Health. 2015; 9(4):277–287. 10.1002/pmh.1310 26337923

[19] Herr NR, Rosenthal MZ, Geiger PJ, Erikson K. Difficulties with emotion regulation mediate the relationship between borderline personality disorder symptom severity and interpersonal problems. Personal Ment Health. 2013; 7:191–202. 10.1002/pmh.1204 24343962

[20] Euler S, Nolte T, Constantinou M, Griem J, Montague P, Fonagy O. Interpersonal Problems in Borderline Personality Disorder: Associations with Mentalizing, Emotion Regulation, and Impulsiveness. J Personal Disord. 2019; 33,427. 10.1521/pedi_2019_33_427 30920937

[21] Gratz KL, Weiss NH, McDermott MJ, Dilillo D, Messman-Moore T, Tull MT. Emotion Dysregulation Mediates the Relation Between Borderline Personality Disorder Symptoms and Later Physical Health Symptoms. J Personal Disord. 2017; 31(4):433–448. 10.1521/pedi_2016_30_252 27322577 PMC5472518

[22] Kells M, Joyce M, Flynn D, Spillane A, Hayes A. Dialectical behaviour therapy skills reconsidered: applying skills training to emotionally dysregulated individuals who do not engage in suicidal and self-harming behaviours. Borderline Personal Disord Emot Dysregulation. 2020;7(3). 10.1186/s40479-020-0119-y 32021690 PMC6993331

[23] Glenn CR, Klonsky ED. Emotion dysregulation as a core feature of borderline personality disorder. J Personal Disord. 2009; 23(1):20–28. 10.1521/pedi.2009.23.1.20 19267659

[24] Ibraheim M, Kalpakci A, Sharp C. The specificity of emotion dysregulation in adolescents with borderline personality disorder: comparison with psychiatric and healthy controls. Borderline Personal Disord Emot Dysregulation. 2017; 4:1. 10.1186/s40479-017-0052-x 28078089 PMC5223469

[25] Chapman AL. Borderline personality disorder and emotion dysregulation. Dev Psychopathol. 2019; 31:1143–1156. 10.1017/S0954579419000658 31169118

[26] Daros AR, Guevara MA, Uliaszek AA, McMain SF, Ruocco AC. Cognitive Emotion Regulation Strategies in Borderline Personality Disorder: Diagnostic Comparisons and Associations with Potentially Harmful Behaviors. Psychopathology. 2018; 51(2):83–95. 10.1159/000487008 29566390

[27] Garnefski N, Kraaij V, Spinhoven P. Negative life events, cognitive emotion regulation and depression. Personal Individ Differ. 2001; 30:1311–1327.

[28] Van Wijk-Herbrink M, Andrea H, Verheul R. Cognitive Coping and Defense Styles in Patients with Personality Disorders. J Personal Disord. 2011; 25(5):634–644. 10.1521/pedi.2011.25.5.634 22023300

[29] Heidari S, Aliloo MM. Comparative Evaluation of Cognitive Emotion Regulation between "B" Personality Disorders and Normal Persons. Procedia—Social and Behavioral Sciences. 2015; 185: 54–60.

[30] Akyunus M. Cognitive aspects of personality disorders: Influences of basic personality traits, cognitive emotion regulation, and interpersonal problems. PhD. Dissertation, Istanbul Sehir University. 2012.

[31] Badoud D, Billieux J, Eliez S, Imhof A, Heller P, Eytan A, et al. Covariance and specificity in adolescent schizotypal and borderline trait expression. Early Interv Psychiatry. 2015; 9(5):378–87. 10.1111/eip.12120 24428891

[32] Sauer C, Sheppes G, Lackner HK, Arens EA, Tarrasch R, Barnov S. Emotion regulation choice in female patients with borderline personality disorder: Findings from self-reports and experimental measures. Psychiatry Res. 2016; 242:375–84. 10.1016/j.psychres.2016.04.113 27344452

[33] Kabat-Zinn J. Full catastrophe living: Using the wisdom of your body and mind to face stress, pain, and illness. New York: Delacorte; 1990.

[34] Fossati A, Porro FV, Maffei C, Borroni S. Are the DSM-IV Personality Disorders Related to Mindfulness? An Italian Study on Clinical Participants. J Clin Psychol. 2012; 68(6):672–683. 10.1002/jclp.21848 22517635

[35] Wupperman P, Neumann CS, Whitman JB, Axelrod SR. The Role of Mindfulness in Borderline Personality Disorder Features. J Nerv Ment Dis. 2009; 197:766–771. 10.1097/NMD.0b013e3181b97343 19829206

[36] Yu M, Clark M. Investigating Mindfulness, Borderline Personality Traits, and Well-Being in a Nonclinical Population. Psychology. 2015;6:1232–1248.

[37] Didonna F, Rossi R, Ferrari C, Iani L, Pedrini L, Rossi N, et al. Relations of mindfulness facets and psychological symptoms among individuals with a diagnosis of Obsessive-Compulsive Disorder, Major Depressive Disorder and Borderline Personality Disorder. Psychol Psychother Theory Res Pract. 2019; 92(1):112–130 10.1111/papt.12180 29575447

[38] Nicastro R, Jermann F, Bondolfi G, McQuillan A. Assessment of Mindfulness with French Version of the Kentucky Inventory of Mindfulness Skills in Community and Borderline Personality Disorder Samples. Assessment. 2010; 17:197–205. 10.1177/1073191110363551 20212075

[39] Shorey RC, Elmquist J, Wolford-Clevenger C, Gawrysiak MJ, Anderson S, Stuart GL. The relationship between dispositional mindfulness, borderline personality features, and suicidal ideation in a sample of women in residential substance use treatment. Psychiatry Res. 2016; 238:122–128. 10.1016/j.psychres.2016.02.040 27086221 PMC4834542

[40] Gratz KL, Rosenthal MA, Tull MT, Lejuez CW. An experimental investigation of emotion dysregulation in borderline personality disorder. J Abnorm Psychol. 2006;115, 850–855. 10.1037/0021-843X.115.4.850 17100543

[41] Chambers R, Gullone E, Allen NB. Mindful emotion regulation: An integrative review. Clin Psychol Rev. 2009; 29(6):560–572. 10.1016/j.cpr.2009.06.005 19632752

[42] Gilbert P, Procter S. Compassionate Mind Training for People with High Shame and Self-Criticism: Overview and Pilot Study of a Group Therapy Approach. Clin Psychol Psychother. 2006; 13:353–79.

[43] Neff KD. The development and validation of a scale to measure Self-Compassion. Self Identity. 2003; 2:223–250.

[44] Sirois FM, Kitner R, Hirsch JK. Self-compassion, affect, and health-promoting behaviors. Health Psychol. 2015; 34(6):661–669. 10.1037/hea0000158 25243717

[45] Vettese LC, Dyer CE, Li WL, Wekerle C. Does self-compassion mitigate the association between childhood maltreatment and later emotion regulation difficulties? A preliminary investigation. Int J Ment Health Addict. 2011; 9(5):480.

[46] Kuo JR, Khoury JE, Metcalfe R, Fitzpatrick S, Goodwill A. An examination of the relationship between childhood emotional abuse and borderline personality disorder features: The role of difficulties with emotion regulation. Child Abuse Negl. 2015; 39:147–155. 10.1016/j.chiabu.2014.08.008 25192957

[47] Aquino Ferreira LF, Pereira FHQ, Benevides AMLN, Melo MCA. Borderline personality disorder and sexual abuse: a systematic review. Psychiatry Res. 2018; 262:70–77. 10.1016/j.psychres.2018.01.043 29407572

[48] Dvir Y, Ford JD, Hill M, Frazier JA. Childhood maltreatment, emotional dysregulation, and psychiatric comorbidities. Harv Rev Psychiatry. 2014; 22(3):149–161. 10.1097/HRP.0000000000000014 24704784 PMC4091823

[49] Linehan M. Cognitive–behavioral treatment of borderline personality disorder. New York: Guilford Press; 1993.

[50] Keng SL, Wong YY. Association among self-compassion, childhood invalidation, and borderline personality disorder symptomatology in a Singaporean sample. Borderline Personal Disord Emot Dysregulation. 2017; 4(1):24. 10.1186/s40479-017-0075-3 PMC5704523 29209501

[51] Gilbert P, Irons C. Focused therapies and compassionate mind training for shame and self-attacking. In: Gilbert P, editor. Compassion: Conceptualisations, research and use in psychotherapy. London: Routledge; 2005. pp 263–325.

[52] Leichsenring F, Leibing E, Kruse J, New AS, Leweke F. Borderline personality disorder. Lancet. 2011;377(9759):74–84. 10.1016/S0140-6736(10)61422-5 21195251

[53] Feliu-Soler A, Pascual JC, Elices M, Martín-Blanco A, Carmona C, Cebolla A, et al. Fostering Self-Compassion and Loving-Kindness in Patients With Borderline Personality Disorder: A Randomized Pilot Study. Clin Psychol Psychother. 2016; 24(1):278–286. 10.1002/cpp.2000 26818533

[54] Southwick SM, Yehuda R, Giller EL. Psychological dimensions of depression in borderline personality disorder. Am J Psychiatry. 1995; 152(5):789–91. 10.1176/ajp.152.5.789 7726321

[55] Scheibner HJ, Daniels A, Guendelman S, Utz F, Bermpohl F. Self-Compassion Mediates the Relationship Between Mindfulness and Borderline Personality Disorder Symptoms. J Personal Disord. 2017; 1–19. 10.1521/pedi_2017_31_331 29120280

[56] Brown KW, Ryan RM, Creswell JD. Mindfulness: Theoretical foundations and evidence for its salutary effects. Psychol Inq. 2007; 18:211–237.

[57] Ebert A, Edel MA, Gilbert P, Brüne M. Endogenous oxytocin is associated with the experience of compassion and recalled upbringing in Borderline Personality Disorder. Depression and Anxiety. 2018; 35(1):50–57. 10.1002/da.22683 28881460

[58] Miklósi M, Martos T, Kocsis-Bogár K, Perczel-Forintos D. A Kognitív Érzelem-Reguláció Kérdőív magyar változatának pszichometriai jellemzői. Psychiatr Hung. 2011; 26(2):102–111. 21653995

[59] Gratz KL, Roemer L. Multidimensional assessment of emotion regulation and dysregulation: development, factor structure, and initial validation of the difficulties in emotion regulation scale. J Psychopathol Behav Assess. 2004; 26:41–54.

[60] Kökönyei Gy, Róbert U, Reinhardt M, Józan A, Demetrovics Zs. The Difficulties in Emotion Regulation Scale (DERS): Factor structure in chronic pain patients. J Clin Psychol. 2014; 70(6):589–600. 10.1002/jclp.22036 24002923

[61] Neff KD. The Self-Compassion Scale is a Valid and Theoretically Coherent Measure of Self-Compassion. Mindfulness. 2016; 7(1):264–276.

[62] Sági A, Komlósi AV, Köteles F. Az Önmagunk Iránt Érzett Együttérzés (Önegyüttérzés) skála magyar változatának pszichometriai jellemzői. Pszichológia. 2013; 33(4):294–312.

[63] Baer RA, Smith GT, Lykins E, Button D, Krietemeyer J, Sauer S. Construct validity of the five facet mindfulness questionnaire in meditating and non-meditating samples. Assessment. 2008; 15:329–342. 10.1177/1073191107313003 18310597

[64] Hedges LV. Distribution Theory for Glass’s Estimator of Effect size and Related Estimators. J Educ Stat. 1981; 6: 2:107–128.

[65] Cohen J. Statistical power analysis for the behavioral sciences. Lawrence Erlbaum Associates; 1988.

[66] Mueller KE, Barton CN. Approximate Power for Repeated-Measures ANOVA Lacking Sphericity. Journal of the American Statistical Association. 1989; Volume 84, No. 406, 549–555.

[67] Mueller KE, LaVange LE, Ramey SL, Ramey CT. Power Calculations for General Linear Multivariate Models Including Repeated Measures Applications. Journal of the American Statistical Association. 1992; Volume 87, No. 420, 1209–1226. 10.1080/01621459.1992.10476281 PMC4002049 24790282

[68] Chapman AL, Law KC. Borderline personality features as a potential moderator of the effect of anger and depressive rumination on shame, self-blame, and self-forgiveness. J Behav Ther Exp Psychiatry. 2015; 46:26–34. 10.1016/j.jbtep.2014.07.008 25194639

[69] Morey LC. Personality Assessment Inventory: Professional manual. Odessa, FL: Psychological Assessment Resources. 1991

[70] Lotfi M, Amini M, Fathi A, Karami A, Ghiasi S. Personality Traits, Emotion Regulation and Impulsive Behaviors in Patients with Borderline Personality Disorder. Practice in Clinical Psychology. 2014; 2(1):27–33

[71] Gutz L, Roepke S, Renneberg B. Cognitive and affective processing of social exclusion in borderline personality disorder and social anxiety disorder. Behav Res Ther. 2016; 87:70–75. 10.1016/j.brat.2016.08.020 27616717

[72] Swannell S, Martin G, Page A, Hasking P, Hazell P, Taylor A, et al. Child maltreatment, subsequent non-suicidal self-injury and the mediating roles of dissociation, alexithymia and self-blame. Child Abuse Negl. 2012; 36(7–8):572–584. 10.1016/j.chiabu.2012.05.005 22858062

[73] American Psychiatric Association. Diagnostic and statistical manual of mental disorders. 5th edition. Washington, DC; 2013.

[74] Choudhury S, Sahoo S, Dash SR. Emotion dysregulation in patients with major depressive disorder and borderline personality disorder. Indian J Public Health Res Dev. 2020; 11(1):624–629.

[75] Baer RA, Smith GT, Allen KB. Assessment of Mindfulness by Self-Report: The Kentucky Inventory of Mindfulness Skills. Assessment. 2004; 11(3):191–206 10.1177/1073191104268029 15358875

[76] Roediger E, Stevens B, Brockman R. Contextual Schema Therapy. Context Press; 2018.

[77] Van Vreeswijk M, Broersen J, Schurink G. Mindfulness and schema therapy: A practical guide. John Wiley & Sons; 2014.

[78] Schanche E, Stiles TC, McCullough L, Svartberg M, Nielsen GH. The relationship between activating affects, inhibitory affects, and self-compassion in patients with Cluster C personality disorders. Psychotherapy. 2011; 48(3):293–303. 10.1037/a0022012 21604900

[79] Castilho P, Costa J, Marôco J, Pinto-Gouveia J, Ferreira C. Validation of the Psychometric Properties of the Self-Compassion Scale. Testing the Factorial Validity and Factorial Invariance of the Measure among Borderline Personality Disorder, Anxiety Disorder, Eating Disorder and General Populations. Clin Psychol Psychother. 2016; 23(5):460–468. 10.1002/cpp.1974 26289027

[80] Auerbach JS, Blatt SJ. Self-representation in severe psychopathology: The role of reflexive self-awareness. Psychoanalytic Psychology, 1996. 13 *(*3), 297–341.

[81] Skodol AE, Bender DS. Why are women diagnosed bordeline more than men? Psychiatr Q. 2003 Winter;74(4):349–60. 10.1023/a:1026087410516 14686459

[82] Grant BF, Chou SP, Goldstein RB, et al. Prevalence, correlates, disability, and comorbidity of DSM-IV borderline personality disorder: results from the Wave 2 National Epidemiologic Survey on Alcohol and Related Conditions. J Clin Psychiatry. 2008; 69:533–545 10.4088/jcp.v69n0404 18426259 PMC2676679

[83] Goodman M, Patil U, Steffel L, et al. Treatment utilization by gender in patients with borderline personality disorder. J Psychiatr Pract. 2010; 16:155–163. 10.1097/01.pra.0000375711.47337.27 20485103

