# Supplementary material for: Emotion regulation, mindfulness, and self-compassion among patients with borderline personality disorder, compared to healthy control subjects
Source: PLoS One. 2021 Mar 17;16(3):e0248409. doi: 10.1371/journal.pone.0248409 (PMC7968662; doi:10.1371/journal.pone.0248409)
Supplement: Attachment — Submitted filename: Review_PONE-D-20-18458.docx [file pone.0248409.s002.docx]

Thank you for the oppurtunity to review this manuscript. While I believe that the topic itself is very important and the clinical sample sizes are quite large, there are at least three major issues that need to be addressed before the manuscript can be accepted:

(1) Lack of clarity and motivation for the comparison of BPD and other PD in the introduction.

In the beginning of the introduction, the authors cite many studies that show that emotion regulation difficulties are already well established as a core of BPD. Why do we need one further study? While I understand that it is a very important task to compare BPD not only to a HC group but also other clinical samples, why the authors choose to compare the group to other PD remains unclear. What is the benefit of this comparison? In addition, given that the group of other PD is very heterogenous and due to comorbidities both groups do not even differ on the amount of several diagnosed personality disorders (see table 1), the comparison becomes methodologically questionable. Who are you really comparing against whom here?

At the same time, the introduction is very lengthy and should be shortened to only include the most relevant information.

(2) PCA

The motivation for the PCA remains unclear. In addition, one could question whether it is a good idea to perform a PCA with such a heterogenous sample. The authors should include measures of instability, such as bootstrapping and cross validation.

(3) Language and formatting

Throughout the manuscript there are many language inconsistencies and some formatting mistakes, for example in the tables. As a reviewer, I have only limited time and cannot point out every language mistake, I highly recommend proof reading by a native speaker specialized in research articles.
