# Supplementary material for: Emotion regulation, mindfulness, and self-compassion among patients with borderline personality disorder, compared to healthy control subjects
Source: PLoS One. 2021 Mar 17;16(3):e0248409. doi: 10.1371/journal.pone.0248409 (PMC7968662; doi:10.1371/journal.pone.0248409)
Supplement: Attachment — Submitted filename: PLOS One Response to reviewers 23_02_2021-1.docx [file pone.0248409.s004.docx]

February 23, 2021

Stephan Doering, M.D.

Academic Editor

PLOS ONE

Manuscript Number: PONE-D-20-18458

Title: Emotion regulation, mindfulness, and self-compassion among patients with borderline personality disorder, compared to healthy control subjects

Dear Professor Stephan Doering,

Thank you very much for your letter, which provided us with the opportunity to revise our manuscript.

Based on the helpful suggestions of the reviewers, we have revised the manuscript carefully. I have enclosed a revised version of the above paper for submission to PLOS ONE. We have addressed the comments raised by the reviewers.

Point-by-point responses to the reviewers’ comments are listed as follows.

- Reviewer 3 Comment 1

You responded to my critique about the other PD group and the PCA by simply omitting both of them. I find this problematic, because it does not answer the question “why do we need one further study?” And leaves you with a very basic design.
In particular, at the end of your introduction, please rewrite the following paragraph:
“We hypothesized that they are less able to use functional emotion regulation, such as being mindfully aware of one's emotions, to label, accept and validate emotions, and to tolerate negative or positive emotion-related distress without non-adaptive reactivity, putting into perspective, positive refocusing, positive reappraisal of the situation and refocus on planning. In the current study, we aimed to investigate whether certain emotion regulation difficulties are specifically characteristic of BPD patients compared to a healthy control group. We also wanted to examine emotion regulation difficulties, adaptive and maladaptive cognitive emotion regulation strategies, mindfulness, and self-compassion in the two groups.”
And please explain how your study is different from previous studies or how, if it is not different, it is still valuable to have additional data.

Response 1

We thank the Reviewer for drawing attention to this very important question. We added a new subsection under 1.6 (Mini-review of the literature) where we explain how or study is different from previous ones:

“Why do we need one further study? As outlined in the Introduction, there are several studies examining emotion regulation difficulties in BPD. However, there are only a few studies comparing adult BPD groups to healthy control participants, and those that exist do not examine CERQ, DERS, FFMQ and SCS simultaneously by analyzing all of their subscales. We prepared a summary of the literature that compares adult BPD and HC groups by using CERQ, DERS, FFMQ and/or SCS (see Table 1). By administering these four questionnaires in the two groups in the current study, we cover a more comprehensive array of emotion regulation strategies than previous studies.”

We also modified the above quoted paragraph:

“In the current study, we aimed to investigate whether a broad range of emotion regulation difficulties are characteristic to BPD patients compared to a healthy control group. We also wanted to examine emotion regulation difficulties, adaptive and maladaptive cognitive emotion regulation strategies, mindfulness, and self-compassion in the two groups. Our study is partly a replication and partly an extension of previous studies.”

- Reviewer 3 Comment 2

“Our study investigated emotion regulation difficulties that are characteristic of Borderline Personality Disorder (BPD), compared to a healthy control group “
Borderline personality disorder should not be capitalized

Response 2.

We thank the Reviewer for this comment. We corrected this mistake.

- Reviewer 3 Comment 3

“In comparison to a healthy control group, BPD patients have a serious problem in the following areas:”
“have a serious problem” is colloquial and cannot be proven from the data.

Response 3.

Thank you for this comment. We rephrased this sentence to the following:

“In comparison to a healthy control group, BPD patients show deficits in the following areas: mindfulness, self-compassion and adaptive emotion-regulation strategies.”

- Reviewer 3 Comment 4

“In our study, we would like to compare emotional dysregulation in the BPD and HC groups in an adult sample by using DERS as a measurement tool for emotion dysregulation.”
Either explain how this is different from previous studies or at least recognize:
“In our study, we would like to replicate previous findings and …”

Response 4.

Thank you for this comment. Please see Response 1.

- Reviewer 3 Comment 5

“between people of cluster B PDs” – omit the blank space between B and P.

Response 5.

Thank you for your comment. We corrected this part to “between people of cluster B personality disorders”.

- Reviewer 3 Comment 6

“We also assumed that adaptive emotion regulation strategies, mindfulness skills, and self-compassion techniques would score higher in the HC group.”
“We also hypothesized that…”

Response 6.

Thank you for this comment. We rewrote the sentence accordingly.

- Reviewer 3 Comment 7

Differences among the BPD and HC groups in terms of their DERS, CERQ, FFMQ and SCS sub-scales were investigated by One-way Analysis of Variance (ANOVA).
If you have only two groups, you do not need an ANOVA to test for differences but a simple t-test would suffice. However, if you want to test for group differences on more than one scale, it could make sense to calculate a multivariate analysis of variance (MANOVA) with all 4 scales or their subscales as dependent measures, instead of correcting for multiple testing. It should not make major differences in the results, however I would advise with your statistician.

Response 7.

Thank you for this comment. We calculated a multivariate analysis of variance.

Page 8.

“Our statistical analyses tested the hypothesis that difficulty of emotion regulation scores are higher in patients with borderline personality disorder than in healthy participants against the null-hypothesis of no difference. The differences between the BPD and HC groups in terms of their DERS, CERQ, FFMQ and SCS sub-scales were investigated by Multivariate Analysis of Variance (MANOVA), and subsequently by post-hoc univariate F-test statistics determined from the MANOVA analysis.

The analyses were conducted based on a hierarchical approach. Specifically, first, in our primary analysis, the total score on each of the four scales of interest was tested. Study group (BPD or HC) was used as the independent variable in the MANOVA, whereas DERS-total, CERQ adaptive emotion regulation total, CERQ maladaptive emotion regulation total, FFMS-total, and SCS-total scales served as dependent variables. Second, in case the primary analyses yielded a significant difference, we conducted post-hoc analyses by determining the univariate F-statistics to examine the differences between the two groups in the subscales of the four scales mentioned above.”

- Reviewer 3 Comment 8

“One of the limitations of our study is that self-administered questionnaires might have distorted the data.”
Please elaborate.

Response 8.

Thank you for this comment. We modified this sentence to the following:

“One of the limitations of our study is that self-administered questionnaires might have distorted the data, because self-awareness and self-reflection are impaired functions in BPD (83).”
